# Ten-year surveys on exposure to waste anaesthetic gases in a large hospital by personal, environmental, and biological monitoring

**DOI:** 10.1093/annweh/wxag059

**Published:** 2026-07-23

**Authors:** Laura Campo, Giulia Colella, Luca Olgiati, Andrea Spinazzè, Luca Boniardi, Francesca Borghi, Giacomo Fanti, Dario Consonni, Domenico Maria Cavallo, Silvia Fustinoni

**Affiliations:** Department of Clinical Sciences and Community Health, Dipartimento di Eccellenza 2023-2027, University of Milan, via Francesco Sforza, 35, Milan 20122, Italy; Laboratory of Toxicology, Fondazione IRCCS Ca’ Granda Ospedale Maggiore Policlinico, Via Francesco Sforza, 35, Milan 20122, Italy; Department of Clinical Sciences and Community Health, Dipartimento di Eccellenza 2023-2027, University of Milan, via Francesco Sforza, 35, Milan 20122, Italy; Laboratory of Toxicology, Fondazione IRCCS Ca’ Granda Ospedale Maggiore Policlinico, Via Francesco Sforza, 35, Milan 20122, Italy; Department of Science and High Technology, University of Insubria, via Valleggio 11, Como 22100, Italy; Department of Clinical Sciences and Community Health, Dipartimento di Eccellenza 2023-2027, University of Milan, via Francesco Sforza, 35, Milan 20122, Italy; Department of Science and High Technology, University of Insubria, via Valleggio 11, Como 22100, Italy; Department of Medical and Surgical Sciences, University of Bologna, via P. Palagi 9, Bologna 40138, Italy; Department of Clinical Sciences and Community Health, Dipartimento di Eccellenza 2023-2027, University of Milan, via Francesco Sforza, 35, Milan 20122, Italy; Occupational Health Unit, Fondazione IRCCS Ca’ Granda Ospedale Maggiore Policlinico, Via Francesco Sforza, 35, Milan 20122, Italy; Department of Science and High Technology, University of Insubria, via Valleggio 11, Como 22100, Italy; Department of Clinical Sciences and Community Health, Dipartimento di Eccellenza 2023-2027, University of Milan, via Francesco Sforza, 35, Milan 20122, Italy; Laboratory of Toxicology, Fondazione IRCCS Ca’ Granda Ospedale Maggiore Policlinico, Via Francesco Sforza, 35, Milan 20122, Italy

**Keywords:** anaesthetic gases, sevoflurane, nitrous oxide, desflurane, surgery, dentistry, exposure assessment, biological monitoring

## Abstract

**Introduction:**

Nitrous oxide, sevoflurane, and desflurane are hazardous chemicals used in surgery and dentistry to anaesthetise or sedate patients. During the use, they can be dispersed into the environment, causing exposure of healthcare workers. This study summarises 10 yrs of surveys in a large hospital in Milan, Italy.

**Materials and methods:**

Annual surveys were conducted from 2015 to 2024. Real-time monitoring and personal monitoring of exposure were performed. Biological monitoring was assessed by urinary nitrous oxide, desflurane, and hexafluoroisopropanol (metabolite of sevoflurane).

**Results:**

101 surveys were performed in 25 operating theatres and 5 dentistry rooms, observing 219 interventions; 383 workers were investigated with 600 paired personal and biological measurements. Sevoflurane was the most used gas (49% alone, 5% with nitrous oxide), followed by nitrous oxide (7%) and desflurane (3% alone, 2% with nitrous oxide); no gas was used in 32% of cases. Median personal exposure for sevoflurane, nitrous oxide, and desflurane was <0.1, 1.4, and <0.1 ppm, with a 95th percentile of 1.2 ppm for sevoflurane in the anaesthesiologist and 428 ppm for nitrous oxide in the dentist. Median urinary hexafluoroisopropanol, nitrous oxide, and desflurane were <0.02 mg/L, <2 µg/L, and <0.3 µg/L, respectively. Real-time monitoring showed peaks of sevoflurane and desflurane during the refill of the vaporiser, the use of a facial mask for the induction, and the tracheal extubation; nitrous oxide peaks were observed during sedation in dentistry. Exposures were higher in paediatric than in adult surgery.

**Conclusion:**

Exposure to sevoflurane and desflurane in general anaesthesia is low; exposure to nitrous oxide in dentistry during sedation may overcome occupational limit values.

What's Important About This Paper This study reports data from 10-yr surveys of occupational exposure to anaesthetic gases (sevoflurane, nitrous oxide, and desflurane) in the healthcare personnel of a large hospital. Exposures were mostly well below the occupational limit values, with a few exceptions for the use of nitrous oxide for sedation in dentistry. Determinants of exposure were being an anaesthesiologist, performing paediatric surgery, and using nitrous oxide for sedation.

## Introduction

Anaesthetic gases are used in surgery and dentistry to anaesthetise and/or sedate patients. Since the beginning of the nineties, the preferred halogenated anaesthetics have been sevoflurane (SEVO) and desflurane (DES) (Becker and Rosenberg 2008). Nitrous oxide (N_2_O) was introduced more than 100 yrs ago as an anaesthetic gas; it is still used in surgery, mostly in combination with the halogenated gases. N_2_O is used alone for patient sedation in dentistry and in children’s first aid, as well as to reduce pain during labour (Becker and Rosenberg 2008; Tobias 2013).

SEVO (CAS no. 28523-86-6) and DES (CAS no. 57041-67-5) (Table S1) are polyfluoro ethers, liquid at room temperature, with a high vapour pressure (PubChem Sevoflurane; PubChem Desflurane). SEVO is the most used anaesthetic gas. They are used for the induction and maintenance of general anaesthesia in surgery in adults; SEVO is the preferred gas for paediatric patients. They are classified by industry as hazardous for humans, as they are suspected of damaging fertility or the unborn child, they cause serious eye irritation and skin irritation, and they may cause drowsiness or dizziness (ECHA Chem Sevoflurane; ECHA Chem Desflurane). DES can cause respiratory irritation. SEVO and DES are rapidly adsorbed and distributed; SEVO is biotransformed in a minor amount (up to 5%) by hepatic metabolism to form hexafluoro isopropanol (HFIP), excreted in urine as glucuronide conjugated (ECHA Chem Sevoflurane; Kharasch et al. 1995), while DES is biotransformed in a minor amount to trifluoroacetic acid, excreted in urine within 24 h from administration. Most SEVO and DES are excreted unchanged through the exhaled air and in urine (GESTIS Desflurane; Gestis Sevofluorane; PubChem Desflurane).

N_2_O (CAS no. 10024-97-2) (Table S1) is used in general anaesthesia and for sedation in dentistry. With respect to toxicity in humans, the harmonised classification of the European Chemical Agency reports that it may damage the unborn child, and it is suspected to cause infertility, may cause drowsiness or dizziness, and may cause damage to organs through prolonged or repeated exposure (ECHA Dinitrogen oxide). N_2_O is mostly excreted unchanged in the exhaled air and in urine (Gestis Nitrous oxide; PubChem Nitrous oxide).

In general anaesthesia, anaesthetic gases are administered with a medical device called a ‘continuous-flow anaesthetic machine’, designed to supply fresh medical gases mixed with an accurate concentration of anaesthetic vapour and to deliver this continuously to the patient at a safe pressure and flow. The machine is connected to the patient's respiratory system, forming a closed system that provides a recirculation of anaesthetic gases and to a scrubber to absorb carbon dioxide emitted from the patient, so that expired gas becomes suitable for re-use. An integrated anaesthetic vaporiser, a reservoir responsible for vaporising the anaesthetic agents, which are in the liquid state at room temperature, controls the concentration at which these vapours are added to the fresh gas flow. When empty, the vaporiser needs to be refilled with the liquid anaesthetics from a bottle (Beaulieu et al. 2013).

Anaesthetic gases can also be provided via facial masks. The facial mask can be used to deliver oxygen and anaesthetics, simply placing it over the patient's nose and mouth to create a seal. This mask is used during sedation, analgesia, and general anaesthesia, and it is removed when the patient is asleep. The facial mask is typically used for anaesthesia induction in paediatric surgery. Nose masks are used for sedation in dentistry and kept in place during the intervention. Masks are equipped with a vapour recovery system to limit the dispersion of gas, but they are open devices that allow the dispersion of gas from both the distribution system and the patient's respiratory system.

During surgical and dentistry use, waste anaesthetic gases can disperse in the environment, thus causing exposure to healthcare personnel (CCOHS 2007; Hoerauf et al. 1997; Herzog-Niescery et al. 2017). To assess the hygienic condition of the workplace and the compliance with limit values, air and biological monitoring can be applied.

To characterise the risk from exposure to waste anaesthetic gases, occupational limit values for N_2_O, SEVO, and DES have been established in some countries, and/or some recommendations are available from agencies (see Table S1) (GESTIS International limit values for chemical Agents Sevoflurane; Desflurane; Nitrogen Oxide; NIOSH 1977; Italian Ministry of Health 1989; ACGIH 2025). To interpret our results, this study considered the occupational exposure limits (OELs) of 2 ppm for halogenated anaesthetic gases and 50 ppm for N_2_O, as a time-weighted average of the exposure. For the biological monitoring, it was used the recommendation of a urinary limit value of 27 µg/L at the end of the exposure/end of shift for N_2_O (Italian Ministry of Health 1989), while for SEVO and DES it was considered the biomarker equivalent concentrations to the occupational limit value of 2 ppm for urinary HFIP, metabolite of SEVO and urinary DES, equal to 0.49 mg/L and 0.9 µg/L, respectively, in samples collected at the end of the exposure (Imbriani et al. 2001; Alessio et al. 2003).

In the present study, we report data collected between 2015 and 2024 on occupational exposure to anaesthetic gases among health-care personnel working in the surgical theatres and outpatient dentistry units of a large Italian hospital. Annual surveys were conducted using continuous environmental monitoring, personal sampling, and biological monitoring. Our objective was to assess compliance with OELs and biological equivalent concentrations, as well as to identify the medical specialities, job tasks, and working practices associated with the highest exposure levels.

## Methods

The surveys were carried out in 25 operating theatres and 5 outpatient dentistry rooms of a large Italian hospital from 2015 to 2024. The operating theatres are all equipped with continuous-flow anaesthetic machines with scavenging systems and with an air exchange rate of 15 changes per hour, as required by the technical guidelines for the authorisation of surgery theatres in Italy (DPR 14/01/97). No area designated for vaporiser refilling was present, and the operation was performed in the operating room. The dentistry rooms are equipped with sedation machines dispensing N_2_O and a system to recover the gas; the air exchange rate is 6 changes per hour.

Each survey began in the morning, typically between 07:00 and 08:00, before the start of the surgery activity. Direct reading real-time monitoring of N_2_O, SEVO, and DES in the environments was performed by placing the direct-reading apparatus in the operating theatre. Time-weighted average personal monitoring was performed, giving each healthcare worker in the room a personal passive sampler. Air and personal monitoring were conducted throughout the entire surgical session, encompassing one or more surgeries. Sampling was, in most cases, concluded by 14:00, corresponding to the end of the shift for most healthcare workers. In a limited number of cases involving prolonged surgical procedures, monitoring duration was extended until approximately 15:30.

Biological monitoring was used to assess urinary N_2_O, DES, and HFIP, a metabolite of SEVO. Urine samples were collected at the end of exposure or at the end of the shift.

During each survey, a time-activity diary was used to report information about the patients, the surgery type and duration, the gases used and their concentration, the technical adopted for induction and anaesthesia, and the different phases of the procedure (eg patient entry into the operating room, induction, intubation, maintenance, extubation, patient exit, and the refill of vaporiser), when present. Personal information about the healthcare personnel was also collected using a questionnaire.

The surveys were carried out in compliance with the Italian occupational health and safety legislation (Decreto Legislativo 81/2008), under the employer's responsibility and in agreement with the occupational physician and the health and safety manager of the hospital.

### Real-time monitoring

Real-time monitoring of airborne N_2_O and halogenated gases was carried out from 2015 to 2022 using the photoacoustic analyser Brüel & Kjær 1302, which was replaced in 2023 by the Innova 1512 Photoacoustic Gas Monitor (LumaSense Technologies GmbH, Germany). Air samples were taken sequentially using an Air Plexer 8 multipoint sampler (Airnova, Padova, IT) equipped with probes (10-m length polyamide tubes), positioned in different locations of the operating room, typically at the position of the anaesthesiologist, behind the head of the patient, and on the wall on the side of the operating bed, at a distance of about 2 to 3 m from the surgical staff. The acquisition frequency was set at approximately one measurement every 90 s for each sampling point, thus allowing to observe the presence of concentration peaks in real-time measurements. The lower quantification limits were 1 ppm for N_2_O and 0.01 ppm for halogenated gases. Measurements were automatically corrected for air humidity and carbon dioxide (CO_2_) concentration, as the main confounding factors. Humidity, air pressure, temperature, and the influence of one gas on the other were compensated. The calibration procedures were performed annually by the company commercialising the equipment (Airnova, Padova, IT), using appropriate calibration standards.

### Personal monitoring and analysis

Personal monitoring of N_2_O, SEVO, and DES was performed using radial diffusion samplers (Radiello, AMS Analitica, Pesaro, Italy with grey diffusive body, code RAD1203, and cartridge code RAD132 containing molecular sieves and coconut charcoal, 35 to 50 mesh) worn by healthcare personnel in the breathing zone (the hemisphere of 30 cm radius extending in front of the face) typically for the whole duration of the surgical session. After sampling, the Radiello cartridges were stored in the proper glass tube, stored at 4 °C, and analysed within the maximum range of cartridge stability (30 d).

At the time of analysis, the analytes were desorbed from the cartridge using a 10 ml mixture of MeOH/H_2_O (4:6) for 10 min at room temperature, according to the instructions given by the producer. The analysis was performed by headspace gas chromatography–mass spectrometry (PAL RSI 120 series 2 autosampler interfaced with a gas chromatograph 8890 Agilent and a mass spectrometer 5977/C Agilent). The injection volume was 1 ml; the incubation was for 45 min at 45 °C under agitation (speed 250 rpm); the injection syringe was kept at 70 °C. The separation was performed using the column Restek Rt-Q-bond (Superchrom, Italy) (30 m, 0.32 mm inner diameter, 0.10 µm film thickness). The quantification was performed using a calibration curve. The limit of quantification (LOQ) was 1 ppm for N_2_O and 0.1 ppm for SEVO and DES. The exposure was calculated as a time-weighted average concentration, taking into consideration the exposure time, temperature, and the specific uptake rate of each analyte.

### Biological monitoring and analysis

At the end of exposure/end of shift, with 2 h as a minimal exposure duration, a spot urine sample was collected for biological monitoring to assess urinary N_2_O and/or DES and/or HFIP. The selection of biological markers and the collection of urine samples were driven by the gases used during the interventions.

For the measurement of urinary N_2_O on site, immediately after urine collection, an aliquot of 10 ml urine was transferred to a 20 ml sealed glass vial containing 800 µl of H_2_SO_4_ at 30% in water, as a preservative, using a disposable syringe. This was done by piercing the septum with the syringe needle, while a second needle was simultaneously placed in the septum for the air outlet.

For the measurement of urinary DES on site, immediately after urine collection, an aliquot of 10 ml urine was transferred to an empty 20 ml sealed glass vial. This was done as described above for N_2_O.

Vials containing urine were then placed upside down at room temperature; once in the lab, they were stored upside down at −20 °C until analysis, which was performed within 1 wk.

For HFIP, once in the lab, an aliquot of 5 ml urine was transferred into a PP tube for urine; samples were stored at −20 °C for up to 30 d before analysis.

For N_2_O and DES analysis, the analytes were sampled directly in the headspace of the storage vial, after equilibrating the vial at 45 °C for 10 min under agitation (speed 250 rpm), in the autosampler oven (PAL RSI 120 series 2). The analysis was performed via GC–MS (chromatograph 8890 Agilent and mass spectrometer 5977/C Agilent). The injection volume was 1 ml; the injection syringe was kept at 70 °C. The separation was performed using the column Restek Rt-Q-bond (Superchrom, Italy) (30 m, 0.32 mm inner diameter, 0.10 µm film thickness). The quantification was performed based on a calibration curve. The LOQ was 2 µg/L for N_2_O and 0.1 µg/L for DES.

For HFIP analysis, 2 ml urine was hydrolysed with 800 µl β-glucuronidase in 0.5 M acetate buffer at pH 5.0 (800 U/ml) overnight at 37 °C. The analysis was performed in the headspace of the vial after equilibrating the vial at 60 °C for 10 min under agitation (speed 250 rpm) in the autosampler oven (PAL RSI 120 series 2). The analysis was performed via GC–MS (chromatograph 8890 Agilent and mass spectrometer 5977/C Agilent). The injection volume was 1 ml; the injection syringe was kept at 70 °C. The separation was performed using the chromatographic column CPVAX 52-CB [30 m, 0.25 mm inner diameter, 0.25 µm film thickness [Superchrom, Italy)]. The quantification was performed versus a calibration curve and in the presence of HFIP-d2 as an internal standard. The LOQ was 0.02 mg/L.

### Statistical analysis

Environmental and personal chemical measurements were described using the median, 5th and 95th percentile, and maximum values. The measurements below the LOQ were substituted with half of the LOQ. The concentrations of air anaesthetic gases and urinary biomarkers were log_10_-transformed to approximately achieve a normal distribution and comparison among workers with different job tasks and among different departments, and between adult and paediatric surgery was performed with ANOVA or *t*-test on log-transformed values; post hoc Bonferroni test was used to perform multiple comparisons.

Since workers may have multiple measurements of urinary biomarkers, to take into account within-subject correlations, we fitted simple random-intercept regression models to assess the association between personal exposure to the 3 anaesthetic gases and the corresponding urinary biomarkers; we estimated slopes, 95% confidence intervals (CI), and *P*-values. We also calculated Pearson's correlation coefficients.

Statistical analyses were performed with IBM software SPSS version 29 and Stata 19.

## Results

### The surveys

In Table 1, the details of the surveys performed during the decennial activity are reported; particularly, 25 operating theatres and 5 outpatient dentistry rooms were surveyed in 101 campaigns. A total of 219 interventions were observed, with a median of 2 interventions (range 1 to 9) for each operating session. Seventeen medical specialities were investigated with 1 or more surveys. Most of the interventions were performed on adult patients (*N* = 198) and a minority on paediatric patients (*N* = 21). The paediatric interventions were related to otolaryngology, gastroenterology, and dentistry, with anaesthesia or sedation.

**Table 1:** Summary of the surveys performed in the different surgery and dentistry departments in the period 2015 to 2024, with details about the number of surveys, type of patient, number and duration of the interventions per surgery session, and anaesthetic gases used.

Medical speciality	Surveys (*N*)	Patient type (*N* = 219)		*N* patients per surgery session (*N*)	Surgery duration (min)	*N* surgeries with						Total *N* surgeries
						N_2_O	SEVO	DES	N_2_O + SEVO	N_2_O + DES	No Gas	
		Adults *N* (%)	Children *N* (%)	Min–max	Median (min–max)	*N* (%)	*N* (%)	*N* (%)	*N* (%)	*N* (%)	*N* (%)	*N* (%)
All	101	198 (90%)	21 (10%)	1 to 9	262 (5 to 480)	16 (7%)	108 (49%)	7 (3%)	12 (5%)	5 (2%)	71 (32%)	219
Breast surgery	3	6 (3)	0	2	270 (250 to 295)	0	4	2	0	0	0	6
Cardiology	2	2 (1)	0 (0)	1	218 (140 to 295)	0	2	0	0	0	0	2
Dentistry with anaesthesia	3	2 (1)	7 (33)	2 to 4	217 (185 to 220)	0	1	1	2	5	0	9
Dentistry with sedation	6	13 (7)	2 (10)	1 to 3	39 (5 to 140)	15	0	0	0	0	0	15
Emergency surgery	1	1 (1)	0	1	120	0	1	0	0	0	0	1
Endocrinology	2	2 (2)	0	1	223 (140 to 240)	0	2	0	0	0	0	2
Gastroenterology	8	10 (5)	3 (14)	1 to 3	338 (150 to 420)	0	12	0	0	0	1	13
General surgery	22	51 (26)	0	1 to 8	253 (110 to 425)	0	30	2	1	0	18	51
Gynecology	13	50 (25)	0	1 to 9	220 (92 to 365)	1	11	2	2	0	34	50
Hepatology	4	4 (2)	0	1	380 (315 to 480)	0	4	0	0	0	0	4
Maxillofacial surgery	2	3 (2)	0	1 to 2	310 (295 to 324)	0	2	0	0	0	1	3
Neurosurgery	1	1 (1)	0	1	351	0	1	0	0	0	0	1
Oculistic	2	4 (2)	0	1 to 3	183 (150 to 215)	0	1	0	0	0	3	4
Otolaryngology	5	6 (3)	9 (43)	2 to 4	210 (150 to 300)	0	8	0	7	0	0	15
Plastic surgery	1	6 (3)	0	6	155	0	4	0	0	0	2	6
Pulmonary surgery	8	11 (6)	0	1 to 2	270 (105 to 390)	0	8	0	0	0	3	11
Urology	15	22 (11)	0	1 to 3	320 (145 to 430)	0	15	0	0	0	7	22
Not recorded	3	4 (2)	0	1 to 2	300 (180 to 375)	0	3	0	0	0	1	4

The most used anaesthetic gas was by large SEVO, followed by N_2_O and DES. Sometimes the halogenated gases were administered in combination with N_2_O. Up to 32% of the observed interventions did not use anaesthetic gases, even if some others in the same operating session did; this was particularly the case for adult surgery, while for paediatric surgery, anaesthetic gases were always used. Several specialities were exclusively using SEVO, such as urology, gastroenterology, and pulmonary surgery. The use of N_2_O was mostly associated with paediatric interventions, with the induction performed by a facial mask; this happened in otolaryngology and in dental surgery, followed by anaesthesia. In dentistry with sedation, N_2_O was administered by a nose mask. The overall median duration of the surgical procedure was 262 min, ranging from 39 min for dentistry with sedation to 380 min for hepatology.

### The workers

Altogether, 383 workers were investigated with 600 paired personal air monitoring and end-of-exposure urine samples (see Table S2). In the investigated decade, each worker was investigated from 1 to 9 times. Workers had a mean age of 40 y. The majority was female (55%), with a higher percentage in comparison to male among anaesthetists, nurses, and auxiliary workers, but not among surgeons (39%) and dentists (17%). Workers were divided into 2 macro categories based on surgery performing anaesthesia, and outpatient dentistry with sedation, using exclusively N_2_O. Most measurements were performed in the surgery category (*n* = 577 and 370 workers), while only 23 measurements in 13 workers were performed in the outpatient dentistry. In the surgery, the job tasks were surgeon (*n* = 140, 38%), anaesthetist (*n* = 93, 25%), surgical nurse (*n* = 50, 14%), nurse (*n* = 63, 17%), and auxiliary personnel (*n* = 24, 6%). In outpatient dentistry, the job tasks were dentist (*n* = 6, 46%), sedation machine operator (*n* = 1, 8%), dentistry nurse (*n* = 5, 38%), and auxiliary personnel (*n* = 1, 8%).

### Personal exposure

In Table 2, personal exposure to anaesthetic gases is summarised. Results are depicted for all workers and for workers divided into surgery and outpatient dentistry, and further divided by job tasks. Data are given as the number of measurements, the percentage of measurements above the LOQ, the median, and the 5th and 95th percentiles.

**Table 2:** Summary of results for personal air monitoring of anaesthetic gases in hospital departments, divided into surgery, all specialities, and outpatient dentistry.

	Job task	Workers	Samplings	N_2_O (ppm)				SEVO (ppm)				DES (ppm)			
		*N*	*N*	*N*	% >LOQ	Median	5th–95th	*N*	% >LOQ	Median	5th–95th	*N*	% >LOQ	Median	5th–95th
**All specialities**	All tasks	383	600	172	90	1.4	<0.5 to 71.9	568	32	<0.1	<0.1 to 0.5	63	46	<0.1	<0.1 to 2.0
**Surgery**	**All tasks surgery**	370	577	149	89	1.0	<0.5 to 33.7	568	32	<0.1	<0.1 to 0.5	63	46	<0.1	<0.1 to 2.0
	Surgeon	140	189	45	89	0.7	<0.5 to 18.2	187	25	<0.1	<0.1 to 0.3	18	39	<0.1	<0.1 to 1.4
	Anaesthetist	93	138	32	88	1.2	<0.5 to 203.2	135	50	0.1	<0.1 to 1.2	14	50	0.13	<0.1 to 6.1
	Surgical nurse	50	94	25	96	1.0	0.5 to 22.7	93	27	<0.1	<0.1 to 0.3	10	60	0.3	<0.1 to 2.0
	Nurse	63	126	37	95	0.9	<0.5 to 33.7	124	31	<0.1	<0.1 to 0.4	19	42	<0.1	<0.1 to 5.0
	Auxiliary	24	30	10	90	1.4	<0.5 to 22.7	29	24	<0.1	<0.1 to 0.6	2	50	0.88	<0.1 to 1.7
**Outpatient dentistry**	**All tasks dentistry**	13	23	23	100	28.3	3.3 to 363.9	0	—	—	—	0	—	—	—
	Dentist	6	10	10	100	80.6	4.2 to 428.0	0	—	—	—	0	—	—	—
	Sedation machine operator	1	6	6	100	28.3	4.5 to 98.0	0	—	—	—	0	—	—	—
	Dentistry nurse	5	5	5	100	14.5	1.8 to 59.1	0	—	—	—	0	—	—	—
	Auxiliary	1	2	2	100	3.3	3.3	0	—		—	0	—	—	—

In surgery, all 3 gases were used, with 149 valid measurements for N_2_O, 568 for SEVO, and 63 for DES. In outpatient dentistry, N_2_O was the only gas used, with 23 valid measurements.

Overall, N_2_O was quantified in 90% of measurements (*n* = 155); the percentage of quantification was 89% in surgery versus 100% in outpatient dentistry. The median and 95th percentile were 1.4 and 72 ppm. Overall, 6.4% of samples exceeded the occupational limit value of 50 ppm. In surgery, the median exposure was 1.0 ppm, and the 95th percentile was 33.7 ppm. The exceedances of the limit value were 2.0%. The anaesthetist was the worker with the highest median and 95th percentile. In outpatient dentistry, the median exposure was 28.3 ppm, and the 95th percentile was 364 ppm. The limit value was exceeded by 34.5% of measurements. The dentist was the most exposed worker with a median of 80.6 ppm and a 95th percentile of 428 ppm.

SEVO was quantified in 32% of measurements (*n* = 182). The median was below the LOQ, and the 95th percentile was 0.5 ppm. Only 1.1% of samples exceeded the limit value of 2 ppm. The anaesthetist was the worker with the highest percentage of quantification (50%), the highest median (0.1 ppm), and the highest 95th percentile (1.2 ppm).

DES was quantified in 46% of measurements (*n* = 29). The median was below the LOQ, and the 95th percentile was 2 ppm; 3.2% of samples exceeded the limit value of 2 ppm. The surgical nurse was the task with the highest percentage of quantification (60%). The auxiliary was the task with the highest median (0.88 ppm), and the anaesthetist was the task with the highest 95th percentile (6.1 ppm).

**Figure 1:**
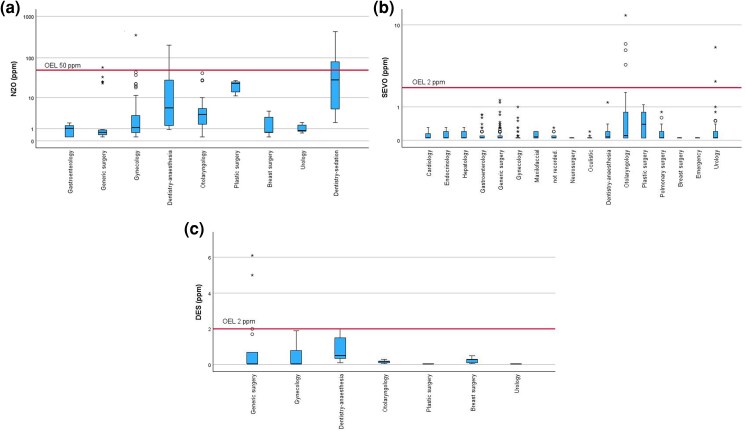
Box plot of the personal exposure to anaesthetic gases by medical speciality. Panel a refers to N_2_O, panel b to SEVO, and panel c to DES. Occupational exposure limits are depicted.

In Fig. 1, personal exposure to N_2_O, SEVO, and DES is reported in box plots in which workers were grouped according to their medical speciality. The comparison among medical specialities shows different exposure to all gases (*P* < 0.05).

N_2_O (panel A) was measured in workers of 7 out of 17 medical specialities, with the highest median exposure in dentistry; median exposure was below the OEL of 50 ppm for all specialities, but a limit value exceedance as high as 35% was observed in dentistry with sedation and 2% in the other surgery specialities. N_2_O exposure was markedly different in the different specialities (*P* < 0.001). Concentrations were higher in dental surgery (both with sedation and with anaesthesia) than in all the other specialities, except for plastic surgery. Levels in plastic surgery were higher than in gastroenterology, general surgery, gynaecology, breast surgery, and urology.

SEVO (panel b) was measured in workers of 17 out of 18 medical specialities. Median exposure as well as the 95th percentile was well below the OEL of 2 ppm, with only 1% exceedance. SEVO exposure was markedly different in the different specialities (*P* < 0.001). Exposure was higher in otolaryngology than in hepatology, gastroenterology, gynaecology, generic surgery, neurosurgery, oculist, pulmonary surgery, breast surgery, and urology. Exposure was higher in plastic surgery than in breast surgery.

DES (panel C) was measured in workers of 7 out of 17 medical specialities, with the highest median exposures in dentistry with anaesthesia. Median exposure as well as the 95th percentile was well below the OEL of 2 ppm, with 3% exceedance. DES exposure was different in the different specialities (*P* = 0.02). Exposure was higher in dentistry with anaesthesia than in plastic surgery and urology.

When all the surgery interventions were divided by type of patient, adults versus children, a higher personal exposure in workers performing paediatric interventions was found for all gases; differences were clear for SEVO (*P* < 0.001), but not for N_2_O and DES. This lack of difference is possibly related to the low number of paediatric patients (34 children versus 138 adults for N_2_O, 8 versus 57 for DES, and 31 versus 537 for SEVO). No mixed surgical session, involving both adults and children, was performed.

### Biological monitoring

Table 3 summarises data on biological monitoring of exposure to anaesthetic gases, specifically urinary N_2_O, HFIP, and DES.

**Table 3:** Summary of results for biological monitoring of anaesthetic gases in hospital departments, divided into surgery, all specialities, and outpatient dentistry.

	Job task	*N* samplings	Urinary N_2_O (µg/L)				HFIP (mg/L)				Urinary dES (µg/L)			
			*N*	% >LOQ	Median	5th–95th	*N*	% >LOQ	Median	5th–95th	*N*	% >LOQ	Median	5th–95th
**All specialities**	**All tasks**	600	77	47	<2	<2–20	551	43	<0.02	<0.02–0.32	35	34	<0.3	<0.3–2.04
**Surgery**	**All tasks surgery**	577	54	37	<2	<2 to 10	551	43	<0.02	<0.02 to 0.32	35	34	<0.3	<0.3 to 2.04
	Surgeon	189	15	20	<2	<2 to 7	178	37	<0.02	<0.02 to 0.28	9	22	<0.3	<0.3 to 0.49
	Anaesthetist	138	13	38	<2	<2 to 12	135	53	<0.02	<0.02 to 0.56	7	43	<0.3	<0.3 to 0.86
	Surgical nurse	94	9	44	<2	<2 to 8	90	36	<0.02	<0.02 to 0.31	6	50	0.42	<0.3 to 2.04
	Nurse	126	15	46	<2	<2 to 10	122	48	<0.02	<0.02 to 0.18	11	36	<0.3	<0.3 to 2.08
	Auxiliary	30	2	50	2	<2 to 3	26	31	<0.02	<0.02 to 0.30	2	0	<0.3	<0.3
**Outpatient dentistry**	**All tasks dentistry**	23	23	70	7	<2 to 118	0	—	—	—	0	—	—	—
	Dentist	10	10	80	9	<2 to 159	0	—	—	—	0	—	—	—
	Sedation machine operator	6	6	83	16	<2 to 118	0	—	—	—	0	—	—	—
	Dentistry nurse	5	5	60	3	<2 to 7	0	—	—	—	0	—	—	—
	Auxiliary	2	2	0	<2	<2	0	—	—	—	0	—	—	—

The total measurements were 77, 551, and 35, of which 47%, 43%, and 34% were above the LOQ, for N_2_O, HFIP, and DES, respectively. Among these, all measured levels of HFIP and urinary DES, along with 54 measurements of urinary N_2_O, were associated with surgical procedures, while only 23 urinary N_2_O measurements pertained to outpatient dentistry with sedation.

Overall, urinary N_2_O was quantified in 47% of measurements (*n* = 36); the percentage of quantification was 37% in surgery versus 70% in outpatient dentistry. The median and 95th percentile were <2 and 20 µg/L. Overall, 2.6% of samples exceeded the concentration of 27 µg/L, recommended as biological equivalent to the occupational limit value of 50 ppm by the Italian Ministry of Health (1989). In surgery, the median and 95th percentile urinary N_2_O were <2 and 10 µg/L, with no exceedance. The anaesthetist was the worker with the highest 95th percentile (12 µg/L). In outpatient dentistry, the median urinary N_2_O was 7, and the 95th percentile was 118 µg/L. The biological equivalent to the occupational limit value was exceeded by 8.7% of measurements. The sedation machine operator was the worker with the highest median urinary N_2_O (16 µg/L); the dentist showed the highest 95th percentile (159 µg/L).

HFIP was quantified in 43% of measurements; however, the median urinary HFIP concentration was below the LOQ (0.02 mg/L) for all job categories. The comparison among the tasks shows the highest concentration in the anaesthetist, with a 95th percentile of 0.56 mg/L. Considering the concentration of 0.49 mg/L, suggested as biological equivalent to the occupational limit value of 2 ppm (Imbriani et al. 2001), we note that 3.4% of measurements exceeded this level.

Urinary DES was quantified in 34% of measurements, with the overall median concentration below the LOQ (0.3 µg/L) and a 95th percentile of 2.04 µg/L. Notably, the surgical nurse had the highest quantification rate (50%) and the highest median concentration of 0.42 µg/L. Considering the concentration of 0.9 µg/L, suggested as biological equivalent to the occupational limit value of 2 ppm (Alessio et al. 2003), we note that 11.4% of measurements exceeded this level.

### Associations between urinary biomarkers and personal exposure

**Figure 2:**
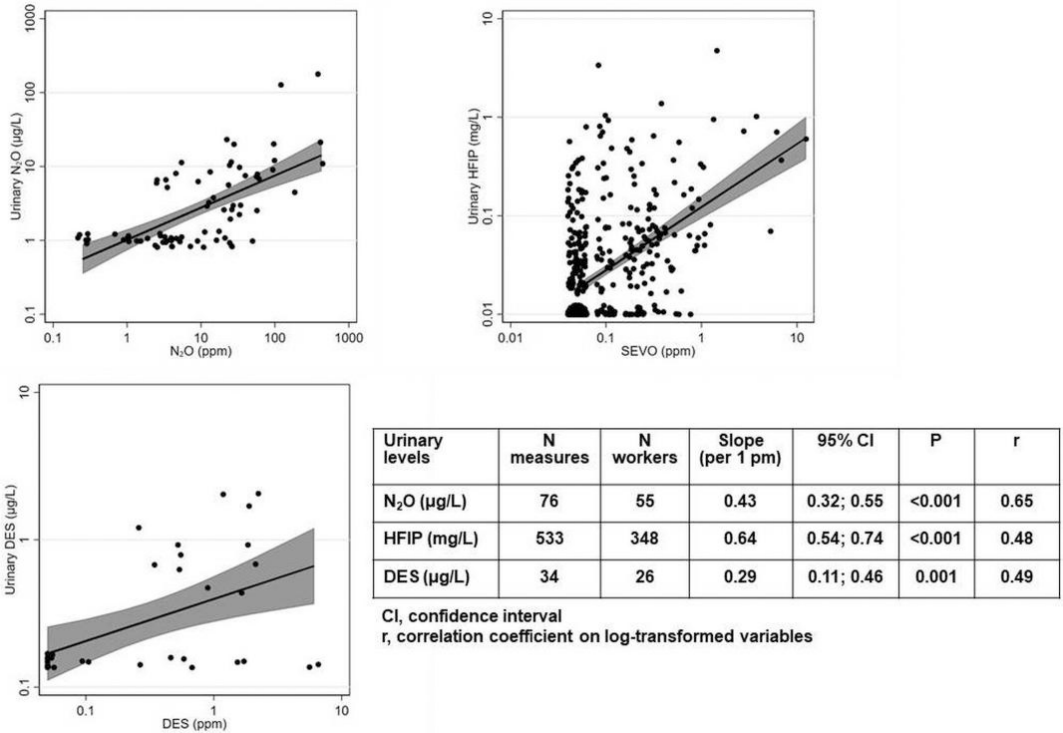
Association between personal exposure to N_2_O, SEVO, and DES and the corresponding urinary biomarkers. The results of random intercept linear regression models (slopes, CIs, and *P*-values) are reported in the table.

We estimated slopes of 0.43 μg/L for N_2_O, 0.64 mg/L for HFIP, and 0.29 for DES, per 1 ppm increase of corresponding personal measurements (Fig. 2). Correlation coefficients ranged from 0.48 to 0.65 (*P* ≤ 0.001).

### Real-time monitoring

**Figure 3:**
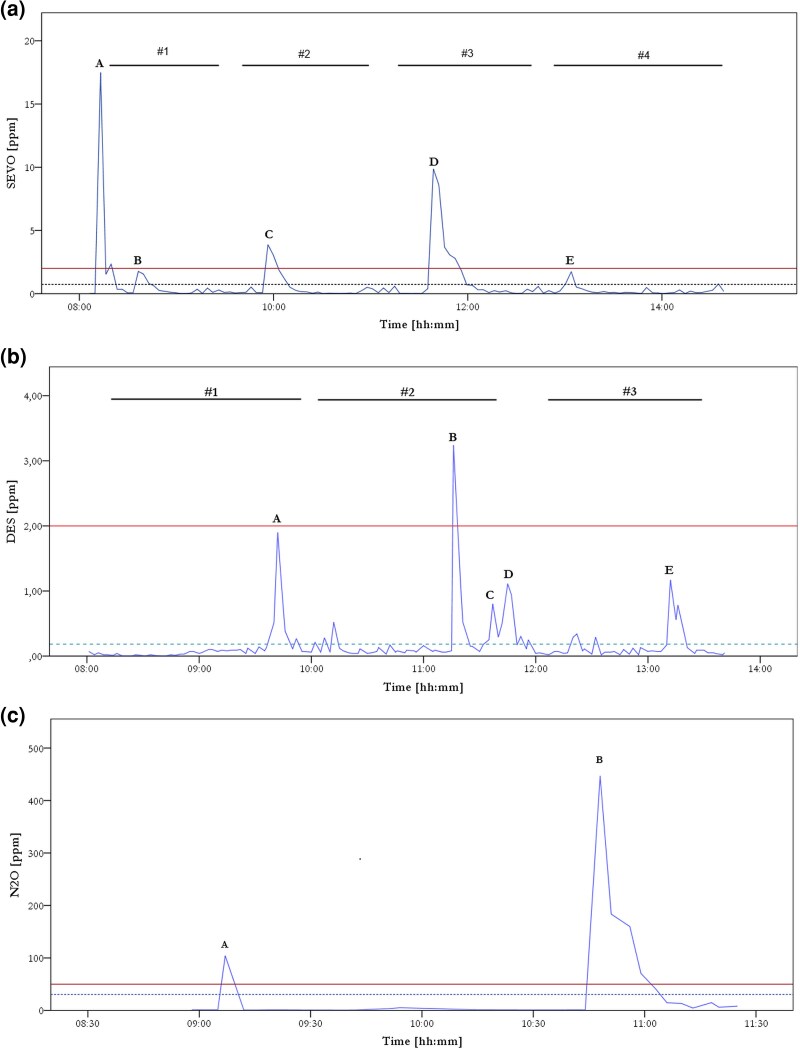
Real-time concentrations of waste anaesthetic gases measured during different procedures in surgery and dentistry. The peaks represent real-time concentrations, the dashed line the mean value, and the solid line the occupational exposure limits (TWA; 2 ppm for SEVO and DES; 50 ppm for N_2_O). Horizontal bars indicate procedure durations. Panel a: SEVO concentrations during 4 paediatric surgeries with inhalation induction; peaks appear during SEVO refilling (a) and induction phases (b to e). Panel b: DES concentrations during 3 surgeries with intravenous induction and maintenance using 5% to 6.5% DES; peaks are linked to gas refills (b, d) and extubation (a, c, e). Panel c: N_2_O concentrations during a dental procedure under conscious sedation; peaks are associated with a system test (a) and gas administration (b).

In Fig. 3, 3 paradigmatic temporal trends of anaesthetic gases in operational sessions for multiple surgery interventions with the use of SEVO (panel 2a) and DES (panel 2b), and one dentistry procedure with the use of N_2_O for sedation (panel 2c) are depicted.

Panel 23 illustrates the temporal trend of SEVO concentrations (solid blue line), the mean measured value (dashed blue line), and the OEL (time-weighted average of 2 ppm, solid red line), recorded during 4 paediatric surgical procedures of otolaryngology (#1 to #4; approximate durations indicated by horizontal black lines). These procedures involved induction with gas dispensed by a facial mask, anaesthesia maintenance with gas dispensed by an intratracheal tube, and extubation. Before the beginning of the session, the vaporiser was filled with liquid SEVO. Major peaks of SEVO are observed in correspondence with the filling of the vaporiser (peak A) and during the induction phase, at the beginning of each procedure (B–E). Notably, the specific nature of these interventions requires the patient's mouth to remain open throughout the surgery, possibly serving as a source of waste anaesthetic gas emissions.

Panel 3B shows the temporal profile of DES concentrations (solid blue line), the mean measured value (dashed blue line), and the time-weighted average of 2 ppm (solid red line), monitored during 3 surgical procedures (#1 to #3; approximate durations indicated by horizontal black lines). These procedures involved intravenous induction of anaesthesia, followed by maintenance with DES (at concentrations between 5% and 6.5%). Distinct concentration peaks are observed during intermittent refilling of the vaporiser performed during the surgical procedures (B and D), as well as at the end of each intervention (A–C–E), corresponding to anaesthetic gas leakage during the extubating procedure and/or emitted by the patient during expiration.

Panel 3C displays the temporal trend of N_2_O concentrations (solid blue line), the measured average value (dashed blue line), and the OEL (time-weighted average of 50 ppm, solid red line), recorded during a single dental procedure performed approximately from 10:45 to 11:05. The intervention was performed on a patient with cognitive disabilities undergoing conscious sedation. N_2_O was administered via a nasal mask equipped with both delivery and scavenging lines for exhaled gases. Notable concentration peaks can be observed during a gas delivery system check (A) and, subsequently, during the actual administration of N_2_O to the patient (B).

## Discussion

The present work summarises 10 yrs of surveys for the assessment of the exposure to anaesthetic gases and the risk for health in the personnel of a large hospital in Milan, Italy.

In our experience, the use of anaesthetic gases is limited to SEVO, DES, and N_2_O; among these, SEVO was used in more than 82% of the interventions in which gases were applied, while the use of DES was limited to 8% of the interventions, although its anaesthetic power is greater than that of SEVO. This pattern of use is common in high-income countries, given the significant greenhouse gas impact of DES. In fact, DES is approximately 2,600 times more potent than carbon dioxide over a 100-yr period, compared to the least potent clinically relevant alternative, SEVO, which is roughly 140 times more potent than carbon dioxide over the same timeframe (Talbot et al. 2025). Following the recognition of its high global warming potential, the use of DES has been subject to increasing restrictions: starting in 2024 in the United Kingdom (National Health Service 2023) and in 2026 in the EU, its use has been or will be strictly limited (European Parliament. Regulation 2024/573). The European Commission's proposal aims to ban DES as an inhalation anaesthetic within the EU, except when strictly necessary on medical grounds (European Parliament. Regulation 2024/573).

For risk assessment for N_2_O, we have used the limit of 50 ppm, as a time-weighted average, recommended by the Italian Ministry of Health (1989), lately confirmed by a document of the Italian insurance institution for the prevention of occupational accidents and diseases (ISPESL 2009). For SEVO and DES, we considered a 2 ppm limit value as the time-weighted average of the exposure period; this is an adaptation of the NIOSH recommendation, dating back to 1977 (NIOSH 1977), indicating 2 ppm as a ceiling level not exceeding 1 h sampling for the halogenated gases. This adaptation was necessary as we applied personal passive samplers to assess the exposure. Indeed, a direct-reading instrument would have been required to verify this compliance, but without the possibility to extend the assessment to all workers, as it was done in our surveys. On the other hand, the NIOSH recommendation was meant to protect the workers’ health from the toxic effects of halothane and other halogenated gases in use in the seventies (Kenna and Jones 1995). The gases in use today, SEVO and DES, are much less toxic than the previous ones, and this is reflected in the specific limit values that have been proposed, with progressively higher concentrations, the last of which are 50 ppm for SEVO and 100 ppm for DES, as an 8-h time-weighted average, recommended by ACGIH (2025) (see Table S1).

For personal air sampling, the Radiello passive sampler was chosen; this is very convenient for its light weight and small volume, reducing the constrain/discomfort of users to the minimum, ensuring the freedom of movement needed by healthcare personnel to achieve the best performance (Radiello; Keller et al. 2022). At the same time, the 3 anaesthetic gases can be sampled at once, with no need for multiple devices; moreover, the radial geometry offers a higher uptake rates in comparison with other commercial passive samplers thus increasing the sensitivity and making the method suitable to assess concentrations as low as 0.1 ppm for SEVO and DES and 1 ppm for N_2_O, when this sampling technique is followed by GC/MS analysis. On the other hand, this sampler, typically worn for the entire surgery session, is suitable to assess time-weighted average exposure, but not short-term exposure.

For the biological monitoring of N_2_O, the unmetabolised N_2_O in urine was measured, and the limit value of 27 µg/L at the end of the exposure/end of shift was used for risk assessment (Italian Ministry of Health 1989). For SEVO, the unmetabolised gas and the urinary metabolite HFIP were used in previous studies with good results and concentrations orders of magnitude higher than that of the unmetabolised SEVO (eg Haufroid et al. 2000; Accorsi et al. 2005; Scappellato et al. 2014). Moreover, considering the low exposures presently observed in operating theatres, it is not surprising that recent studies (Herzog-Niescery et al. 2020; Selke et al. 2023) could not measure urinary SEVO, as it was always below the analytical quantification limit, but could assess HFIP in the same individuals. Based on these experiences and given the low exposures present in our operating theatres, HFIP was selected as the biomarker of choice. An additional advantage of using HFIP over urinary SEVO is its stability, so no precaution is needed for sample collection, and the sample can be stored for months without loss of the analyte. To perform risk assessment, the concentration of HFIP of 0.49 mg/L was used as a biomarker equivalent to the occupational limit value of 2 ppm, as previously proposed (Imbriani et al. 2001). For DES, a single study performed the biological monitoring of exposure in healthcare workers and proposed the measurement of urinary DES (Alessio et al. 2003), showing a good correlation between the urinary DES and personal exposure to DES. In this study, a biomarker equivalent to the occupational limit value of 2 ppm of 0.9 µg/L (Alessio et al. 2003) was proposed, and we used this value to perform risk assessment.

As summarised in Table 1, 101 surveys were performed in 17 medical specialities. Important differences were found comparing exposure in the different specialities, for any gas. Higher concentrations were noticed in otolaryngology and dentistry (Fig. 1) (*P* < 0.05). This may be associated with the use of a mask for the induction of anaesthesia in children, who are among the treated patients in these specialities. The use of a facial mask is performed when the induction with drugs is not feasible due to difficulties in the insertion of the venous access or for other clinical reasons, as it may often be the case in children's surgery. Indeed, paediatric surgery is associated with the highest exposure. This result is in line with previous experiences, where it was shown that the use of a mask is prone to dispersion of gas, due to poor patient compliance and/or to a not good fitting of the mask to the face of the patient (Byhahn et al. 2001; Herzog-Niescery et al. 2017). On the other hand, also in adults, the exposure seems to be increased in otolaryngology; this may be associated with the surgery site and the dispersion of gas through the open mouth.

Considering the surgery, the median time-weighted average exposure in the investigated healthcare personnel was 1.0 ppm for N_2_O and <0.1 ppm for SEVO and DES (see Table 2), well below the limit value of 2 ppm. Higher concentrations were found as 95th percentiles with concentrations of 33.7, 0.5, and 2 ppm for N_2_O, SEVO, and DES (Table 2). These results highlight a very low exposure, even if with some differences among different gases. The lowest exposure was found for SEVO; this gas is mostly used in closed circuit, ie during maintenance of anaesthesia with gas administered with a tracheal tube. This condition is associated with no or minimal gas dispersion. DES is similarly used, but the higher percentage of gas in the mixture (up to 11% in oxygen versus up to 3% for SEVO) can explain the higher exposure observed. N_2_O may be dispersed during the use of the facial mask in the induction of anaesthesia in paediatric surgery or when there is a need to sedate a poorly collaborative patient; both conditions are associated with a dispersion of gas.

Comparing the different job tasks, we confirm findings of previous works (Dehghani et al. 2021) showing that, for SEVO and N_2_O, the aesthetician is the job with the highest exposure, as expected based on its direct handling of anaesthetics. Nevertheless, this exposure is very low, especially for SEVO, with a median concentration of 0.1 ppm. Differently, for DES, the surgical nurse is the job task with the highest median exposure of 0.3 ppm.

In outpatient dentistry (Table 2), the situation is different; N_2_O median exposure was 28.3 ppm, with 95th percentile as high as 364 ppm. About 35% of measurements exceeded the limit value, with the dentist as the most exposed job task, with a median and 95th percentile of 80.6 and 428.0 ppm. This confirms that the use of the nose mask to sedate patients with N_2_O, even if equipped with a recovery system, is a relevant source of exposure, especially for the workers who are closer to the patient (Zaffina et al. 2019). Surely, the high concentration of N_2_O in the sedation mixture (from 30% to 60% N_2_O in O_2_ for adults and from 50% to 70% for children) contributes to this exposure.

The results of biological monitoring are shown in Table 3. Overall, the percentage of quantifiable samples was 47% for N_2_O, 43% for HFIP, and 34% for DES. For N_2_O, a higher quantification percentage was observed in outpatient dentistry (70%) than in surgery (37%), with median concentrations of 7 and <2 µg/L, respectively. Both are well below the biological limit value of 27 µg/L recommended by the Italian Ministry of Health (1989), but an 8.7% exceedance was found in outpatient dentistry, with the dentist and the sedation machine operator as tasks with the highest exposure. For SEVO, the median HFIP urinary concentration was <0.02 mg/L for all tasks, with the highest quantification percentage and 95th percentile, 53% and 0.56 mg/L, respectively, in the anaesthetist. Considering the proposed biological equivalent to the limit value of 2 ppm of 0.49 mg/L (Imbriani et al. 2001), only 3.4% of workers exceeded this concentration, all anaesthetists. For DES, the median urinary concentration was <0.3 µg/L for all tasks except for the surgical nurse, with a median of 0.42 µg/L, a 95th percentile of 2.04 µg/L, and a highest quantification percentile of 50%. Considering the proposed biological equivalent of 0.9 µg/L (Alessio et al. 2003), about 11% of samples showed exceedances.

The results of biological monitoring are in good concordance with those of personal air monitoring, especially for SEVO. In outpatient dentistry, the percentage of exceedance was much higher considering the personal air monitoring than biological monitoring, with exceedances in 35% versus 6% of samples. This may be related to the mode of exposure, which, in dentistry sedation, is characterised by high peaks with short duration; it may be speculated that this is associated with a less than proportional absorption of anaesthetic, therefore leading to a lower-than-expected internal dose.

We found strong increasing biomarker levels with increasing exposure (Fig. 2), as expected; this supports the use of biomonitoring for exposure assessment. In comparison with previous studies, however, the correlations are weaker; this is probably explained by the lower exposure levels observed in our surveys.

The use of real-time monitoring offers a valuable complementary insight into exposure trends and allows the characterisation of temporal concentration patterns, as well as the identification of specific activities or procedural variables associated with exposure peaks. For surgery, as shown for example in Fig. 3a and b, we identified several situations causing exposure peaks, the most common of which are related to: the use of the facial mask for dispensing anaesthetic gases in the induction phase, the refill of the vaporiser with liquid anaesthetics, and the post-surgery extubation of the patient. The identification of these situations allowed us to offer recommendations for risk management, such as the purchase of facial masks with different sizes to improve the adherence to the face of children, and the need to organize the refill of vaporiser before and/or after the surgery section, to have the possibility to perform this operation with care, avoiding the dropping of liquid and limiting the number of by standing workers. Additionally, the refilling system has improved over the years, progressing from simple bottles to new bottles equipped with an adapter specifically designed to fit the vaporiser. Nevertheless, we notice that the peaks of gas were of short duration and efficiently resolved by the ventilation system in place in the rooms. In fact, a high air exchange rate (*n* > 15 changes per hour) is compulsory required by the technical guidelines for the authorisation of surgery theatres in Italy (Decreto Presidente della Repubblica 14 gennaio 1997).

A quite different picture was found in dentistry. The use of N_2_O for sedation of non-collaborative patients, performed by nose mask, is prone to a huge dispersion of gases, even if the recovery system is in place (see Fig. 3c). This is due to the gas emitted by the patient's mouth, which serves as a source (Table 3), while the nose mask is flowing the gas. Moreover, the outpatient clinics for dentistry are not equipped with the same efficient ventilation systems in place in the surgery rooms (6 air changes per hour). Overall, the dispersion can reach relevant concentrations of N_2_O, which is particularly impacting the dentist and the nearby sedation machine operator (Table 3; Fig. 3c). This dispersion may be associated with exposure exceeding the occupational limit value. On the other hand, these dentistry interventions on non-collaborative patients are relatively short in duration and not performed daily, but are typically scheduled once a week, therefore reducing the frequency of exposure. As mitigation measures to reduce exposure in this setting, a procedure for the use of N_2_O and a focus group on the risk for health of N_2_O were implemented. In fact, it was noticed that, while anaesthetists are aware of the toxic effects of the gases and educated to minimise the dispersion, dentists are less conscious.

One limitation of our study is related to the duration of personal air sampling, typically 4 to 6 h, which is much longer than 1 h, maximum duration to verify compliance with a 2 ppm ceiling value for halogenated anaesthetic gases, as recommended by NIOSH. Our sampling time was driven by the use of passive samplers, whose advantages are associated with performing personal monitoring and achieving a high degree of compliance, with the inclusion of all workers in the investigation. A second limitation is associated with the minimum exposure duration of 2 h adopted to consider the surveys valid; we acknowledge that this is much shorter than a typical work shift of 8 h and that this can negatively affect the results of biological monitoring. In our surveys, the median duration of a surgery session was more than 4 h (262 min), and a significantly shorter duration was found only in dentistry with sedation and in emergency surgery (see Table 1). Moreover, the absorption and distribution of anaesthetic gases in the human body is very fast and previous studies with a protocol similar to ours showed good correlations between urinary biomarkers and personal exposure, both considering the unmetabolised gases and the metabolite HFIP (eg Accorsi et al. 2005; Scapellato et al. 2014). A third limitation is associated with the procedures that did not use anaesthetic gases. They were performed in the same surgery sessions as those using gases; they were included in the dataset, and the effect of surgeries performed without gases could not be determined.

## Conclusions

Our decade-long monitoring across operating rooms and dental settings indicates that, on environmental (personal air) measurements, exposures to SEVO and DES are generally low and remain well below OEL values, with exceedances rare and procedure-specific. Biological monitoring corroborated these findings: median biomarker levels were low, and only a small fraction exceeded biological guidance values, typically in staff most proximal to source events. In contrast, N_2_O during dental sedation consistently showed higher ambient levels, with frequent exceedances in facilities lacking effective scavenging and ventilation. Here, biomonitoring showed fewer exceedances than environmental data, consistent with high, but of short duration, peaks that elevate short-term air concentrations but contribute less to integrated biological dose. Procedure-linked peaks were observed (most notably during mask induction and vaporiser refilling), more pronounced in paediatric procedures, underscoring opportunities for targeted prevention. These findings support prioritising technical upgrades and routine performance checks for scavenging and ventilation in outpatient dental clinics, alongside standardised protocols for low-dispersion induction, leak testing, and off-line vaporiser refilling in operating rooms. Collectively, the evidence suggests that current practices ensure low halogenated anaesthetic exposures in surgery, while N_2_O in dentistry remains a prevention priority.

## Supplementary Material

wxag059_Supplementary_Data

## Data Availability

All data generated during this study are presented as descriptive statistics in the main manuscript and its Materials. Raw data cannot be shared publicly in order to protect the privacy of the participating hospital and healthcare personnel.
