# Supplementary material for: Ten-year surveys on exposure to waste anaesthetic gases in a large hospital by personal, environmental, and biological monitoring
Source: Ann Work Expo Health. 2026 Jul 23;70(6):wxag059. doi: 10.1093/annweh/wxag059 (PMC13394691; doi:10.1093/annweh/wxag059)
Supplement: wxag059_Supplementary_Data [file wxag059_supplementary_data.pdf]

Supplementary material

TEN-YEAR SURVEYS ON EXPOSURE TO WASTE ANAESTHETIC GASES IN A LARGE HOSPITAL BY PERSONAL, ENVIRONMENTAL, AND BIOLOGICAL MONITORING

Laura Campo[1,2], Giulia Colella[1], Luca Olgiati[2], Andrea Spinazzè[3], Luca Boniardi[1], Francesca Borghi[3,4], Giacomo Fanti[1], Dario Consonni[5], Domenico M. Cavallo[3], Silvia Fustinoni[1,2]

[1]Department of Clinical Sciences and Community Health, University of Milan, Milan, Italy

[2]Laboratory of Toxicology, Fondazione IRCCS Ca' Granda Ospedale Maggiore Policlinico, Milan, Italy

[3]Department of Science and High Technology, University of Insubria, Como, Italy

[4]Department of Medical and Surgical Sciences, University of Bologna, Bologna, Italy

[5]Occupational Health Unit, Fondazione IRCCS Ca' Granda Ospedale Maggiore Policlinico, Milan, Italy

Supplementary Table 1. Summary of characteristic of the most used anaesthetic gases, national occupational limit values, and agency recommendations.

Substance	CAS n	Molecular weight	Molecular formula	Country/agency	Occupational Exposure Limit values-	
					8-hour time weighted average	Short term (15-minute)
					ppm	ppm
Nitrous oxide N_2O	10024-97-2	44	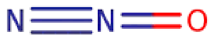	Australia, Canada -Ontario, New Zealand	25	-
				Belgium, Canada-Quebec, Denmark, Ireland, Israel, Norway, Singapore, Spain, Italy	50	100 (Denmark)
				Austria, Finland, Germany, South Africa, Sweden, Switzerland, UK	100	200/400
				ACGIH recommendation, 2025	50	-
				NIOSH recommendation, 1977	25	
				Italian Ministry of Health recommendation, 1989	50	
Sevoflurane SEVO	28523-86-6	205	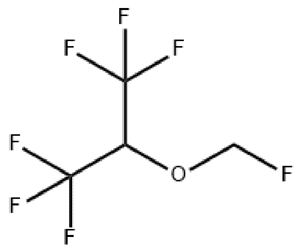	Israel		2
				Norway	5	
				Austria, Finland, Sweden	10	20
				ACGIH recommendation, 2025	50	
Desflurane DES	57041-67-5	168	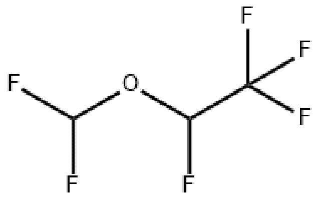	Denmark, Norway	5	10 (Denmark)
				Finland, Sweden	10	20
				Poland	125 mg/m^3	
				ACGIH recommendations, 2025	100	

Halogenated anaesthetic gases	-	-		NIOSH recommendation, 1977		alone: 2 as ceiling together with N_2O: 0.5 *
				Italian Ministry of Health recommendation, 1989		2

*this refers to a ceiling level not exceeding 1-hour sampling.

Supplementary Table 2. Personal characteristics of the investigated workers and number of samplings in the job tasks divided in surgery and dentistry.

		Sampling (N)	Workers (N)	Female (N)	Male (N)	Age (y) mean (min-max)
**All specialties**	**All tasks**	**600**	**383**	**211**	**172**	**40 (21-66)**
**Surgery**	**All surgery**	**577**	**370**	**205**	**165**	**40 (21-66)**
	Surgeon	189	140	54	86	43 (24 - 67)
	Anaesthetist	138	93	54	39	40 (25 - 66)
	Surgical nurse	94	50	39	11	41 (25 – 61)
	Nurse	126	63	43	20	43 (21 - 61)
	Auxiliary worker	30	24	15	9	45 (25 – 58)
**Outpatient dentistry**	**All dentistry**	**23**	**13**	**6**	**7**	48 (27-59)
	Dentist	10	6	1	5	45 (27 – 59)
	Sedation machine operator	6	1	0	1	40
	Dentistry nurse	5	5	4	1	51 (41 – 55)
	Dentistry Auxiliary worker	2	1	1	0	55
